# Combined MediaPipe and YOLOv5 range of motion assessment system for spinal diseases and frozen shoulder

**DOI:** 10.1038/s41598-024-66221-8

**Published:** 2024-07-10

**Authors:** Weijia Zhang, Yulin Li, Shaomin Cai, Zhaowei Wang, Xue Cheng, Nutapong Somjit, Dongqing Sun, Feiyu Chen

**Affiliations:** 1 School of Mathematical Information, Shaoxing University, Shaoxing, China; 2 Department of AOP Physics, University of Oxford, Oxford, UK; 3 Institute of Artificial Intelligence, Shaoxing University, Shaoxing, China; 4 Key Laboratory of Artificial Intelligence Multi-Dimensional Application Research, Shaoxing, China; 5 School of Medicine, Shaoxing University, Shaoxing, China; 6 Department of Neurology, Shaoxing People’s Hospital, Shaoxing, China; 7 School of Electronic and Electrical Engineering, University of Leeds, Leeds, UK

**Keywords:** Spinal diseases, Frozen shoulder, MediaPipe, YOLOv5, Computer science, Information technology

## Abstract

Spinal diseases and frozen shoulder are prevalent health problems in Asian populations. Early assessment and treatment are very important to prevent the disease from getting worse and reduce pain. In the field of computer vision, it is a challenging problem to assess the range of motion. In order to realize efficient, real-time and accurate assessment of the range of motion, an assessment system combining MediaPipe and YOLOv5 technologies was proposed in this study. On this basis, Convolutional Block Attention Module (CBAM) is introduced into the YOLOv5 target detection model, which can enhance the extraction of feature information, suppress background interference, and improve the generalization ability of the model. In order to meet the requirements of large-scale computing, a client/server (C/S) framework structure is adopted. The evaluation results can be obtained quickly after the client uploads the image data, providing a convenient and practical solution. In addition, a game of "Picking Bayberries" was developed as an auxiliary treatment method to provide patients with interesting rehabilitation training.

## Introduction

In the Asian region, due to complex lifestyle habits and the ever-increasing work-related stress, musculoskeletal disorders have become a prevalent concern. Among these disorders, the issues of particular concern are spinal diseases and frozen shoulder, given their seriousness and prevalence in the field. The number of individuals in China afflicted by spine and shoulder pain is nearly 5000 per 100,000 people, and the annual incidence rate ranks within the top three globally, reaching a high of 1037.7 per 100,000 individuals^1^.

Spinal diseases refer to health issues that affect the spine and surrounding structures. The most common symptom is back pain, but other symptoms can include headaches, blurry vision, difficulty standing upright, which may hinder daily activities and, in severe cases, lead to paralysis. Poor posture, such as prolonged desk work in the same position or slouching, increases the risk of developing these conditions. Improving posture, engaging in appropriate exercise, and avoiding excessive fatigue can help alleviate symptoms to some extent and prevent the condition from worsening.

On the other hand, frozen shoulder is an inflammatory condition that affects the tissues surrounding the shoulder joint. It typically presents with shoulder pain and stiffness, leading to limited range of motion in the shoulder joint. This condition may result from factors such as synovitis, capsular fibrosis, and prolonged immobility. Frozen shoulder can impede daily activities such as combing hair, getting dressed, or raising the arm. Early diagnosis and treatment of frozen shoulder are crucial to alleviate pain, restore shoulder function, and prevent further damage. Treatment modalities may include medication therapy and rehabilitative exercises.

Traditional assessment methods for spinal diseases^2^ and frozen shoulder^3^ heavily rely on manual observation and subjective judgment by doctors, resulting in lengthy assessment time and high cost. Recently, there is a strong push to substitute traditional inverse kinematics formulas for human models with deep learning techniques^4,5^. MediaPipe provides an advanced pose estimation framework with robust motion analysis capabilities for the accurate tracking and analysis of patients’ postures and movements^6^. MediaPipe is utilized in research concerning pose estimation and fall detection in elderly individuals^7^. It provides a valuable tool for monitoring seniors' movements, enabling fall detection and proactive assistance. Ardra Anilkumar et al.^8^ proposed an algorithm and study for joint pose estimation in the field of computer vision. This method enables the real-time identification of inaccurate body poses for users to correct errors. Region-based convolutional neural network (R-CNN) introduced the concept of using deep convolutional neural networks (CNN) for detection by proposing to extract features from region proposals^9^. Fast R-CNN improved upon this by introducing the region of interest (ROI) pooling layer to share feature extraction among proposals^10^, and Faster R-CNN further advanced the field by introducing the region proposal network (RPN) to automate proposal generation^11^. Additionally, YOLOv5^12^ is an extension of You Only Look Once (YOLO) series^13–16^. YOLOv5 is an efficient object detection algorithm that can quickly and precisely capture anomalies in target^17^. YOLOv5 performs exceptionally well in complex backgrounds, suitable for object detection, pose detection, and recognition^18^. Hung-Cuong Nguyen et al.^19^ introduced high-precision 2D keypoint and human pose estimation method by combining YOLOv5 and HRNet. Mou, Fangli et al.^20^ proposed a practical approach for the complex task of robotic cable insertion. Du et al.^21^ introduced a multi-task computer vision-based approach for assessing neck exercise, which can server as a substitute for physician-guided rehabilitation and evaluation, particularly beneficial for adolescents.

This paper proposes integrating MediaPipe with YOLOv5 for Asian human pose detection. YOLOv5 performs real-time object detection and labeling, while MediaPipe tracks skeletons and localizes poses, addressing challenges like insufficient feature extraction and background interference. An improved attention mechanism enhances model performance. To address challenges such as hardware demands and economic pressures associated with large-scale computation, this article employs a Client/Server (C/S) architecture. Additionally, a "picking bayberries" game is also developed for rehabilitation assistance.

## Methodology

### Technical principles of the comprehensive motion assessment

**Figure 1:**
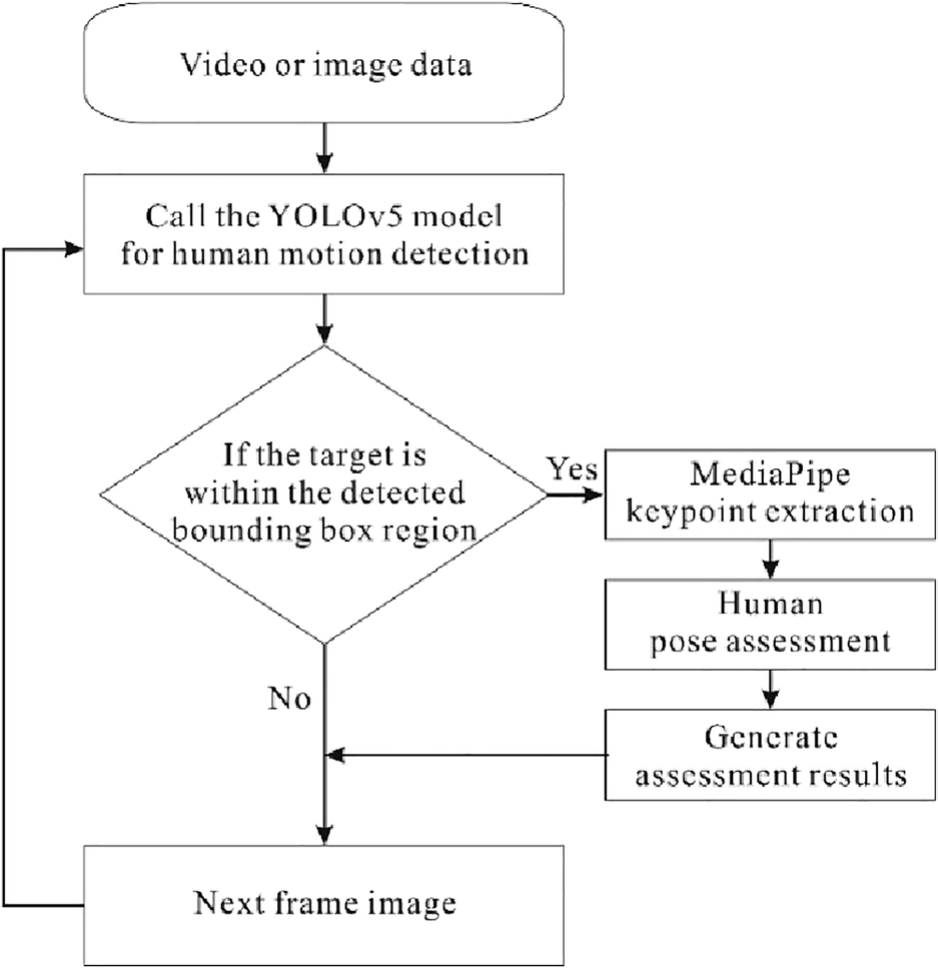
Working principle of the range of motion assessment.

The article introduces an assessment system that combines MediaPipe and YOLOv5 to assess the range of motion related to spinal diseases and frozen shoulder. To facilitate processing and analysis, the system needs to run on a server to ensure sufficient computing resources for rapid handling and response to input data. Additionally, the server should support the scalability and deploy ability of the system, enabling its application in various scenarios and requirements. The system is capable of receiving and processing input video or image data, effectively detecting and evaluating human movement. Its primary fusion method is shown in the Fig. 1. The system first uses YOLOv5 to detect whether the target is within the bounding box, in order to determine the regions of interest. This helps reduce the computational complexity for subsequent processing, as only the target regions are further analyzed, rather than the entire image. This optimization enhances the system's response time while reducing the false positive rate, making target recognition and tracking more reliable and efficient. If the target is detected, then our posture system, based on MediaPipe, is employed to extract keypoints for pose assessment and generate corresponding results. If the target is not within the bounding box, the system skips to analyze the next image. Through this approach, we achieve effective detection and assessment of motion, providing efficient solutions for related fields.

### Posture estimation based on MediaPipe

**Figure 2:**
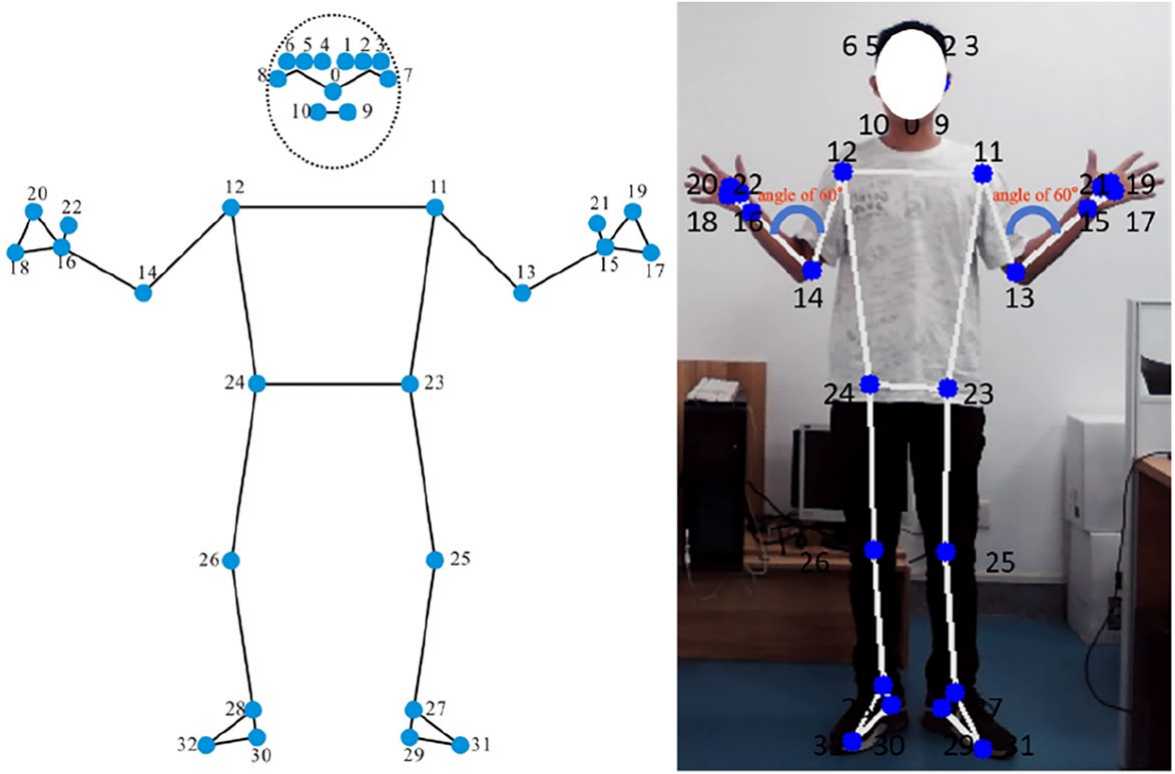
Illustration of MediaPipe keypoints and skeletal lines.

MediaPipe is an open-source machine learning framework primarily used for human pose estimation. MediaPipe employs CNN to achieve human pose estimation, which is a deep learning algorithm that learns patterns and features within images through extensive data training^22^. To commence, the input image is channeled into the MediaPipe library to facilitate the identification of keypoints on the user’s body. The output is a list of coordinates in the X, Y, and Z axes for 33 main body keypoints. In Fig. 2, these landmarks represent the major joints and positions of the human body. These keypoints can be used to estimate the human structure and orientation in a given image or video stream in real-time^23^. MediaPipe is a powerful tool for posture detection and tracking, with wide applications among individuals of different races and cultural backgrounds, including Asians. By combining deep learning technology and computer vision, we can accurately identify the body posture of Asian individuals, including limb positions, joint angles and dynamic body changes.

### Object detection models

#### The working principle of Faster R-CNN

**Figure 3:**
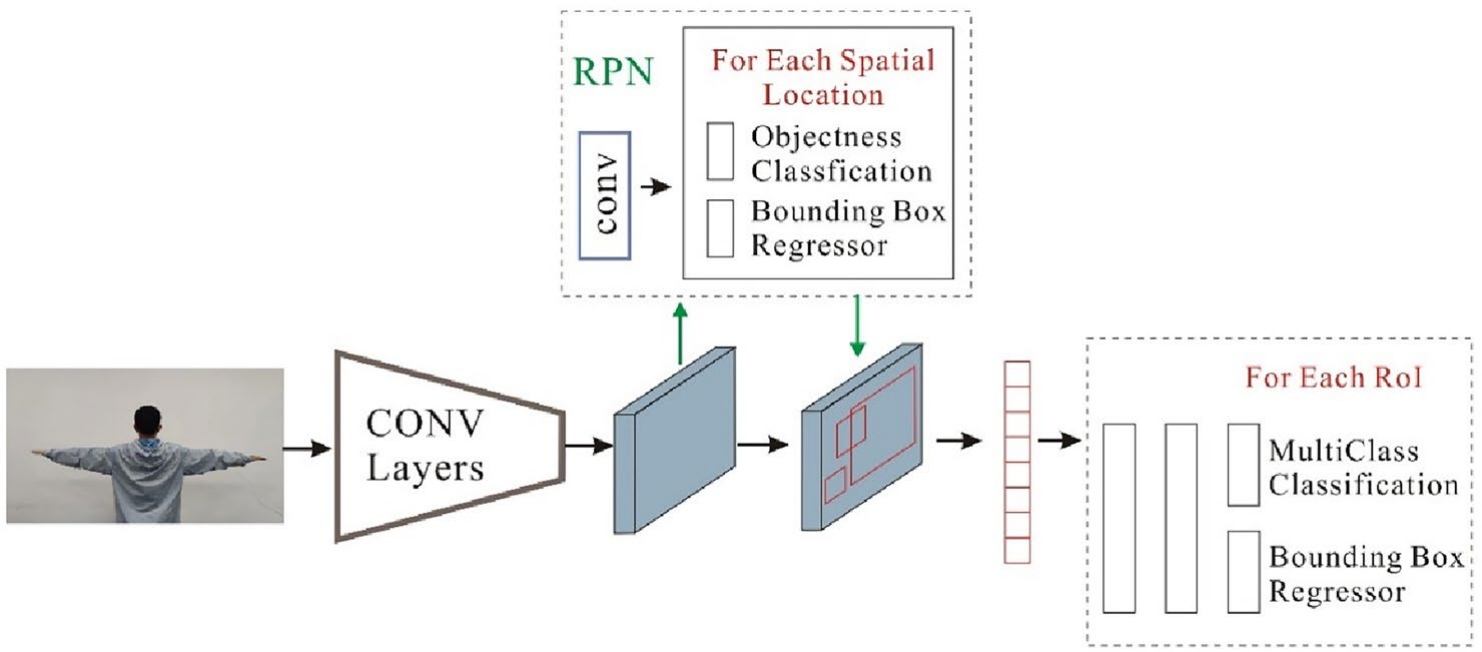
Faster R-CNN network architecture.

Faster R-CNN, an advanced object detection framework, shares a core architecture with Fast R-CNN, but introduces than Region Proposal Network (RPN). It begins by processing an input image with a CNN to extract features and create a feature map. Anchors, predefined bounding boxes of various sizes and aspect ratios, cover the objects. The RPN, another CNN, has two branches: one handles object classification for each anchor, and the other acts as a bounding box regressor, adjusting box boundaries. Region proposals from the RPN are projected onto the feature map, and Rol pooling extracts features for each proposal. The efficient architecture enable precise object detection and classification, making Faster R-CNN a robust tool. As depicted in Fig. 3, we conducted operational runs of Faster R-CNN using our image data.

#### The working principle of YOLOv5

YOLO is a real-time object detection system and neural network model used in computer vision^24^. This algorithm performs forward propagation on the input image by using CNN to directly predict the positions and categories of objects present in the image^25^. It achieves high speed and accuracy in object detection and can handle objects of varying sizes and types.

**Figure 4:**
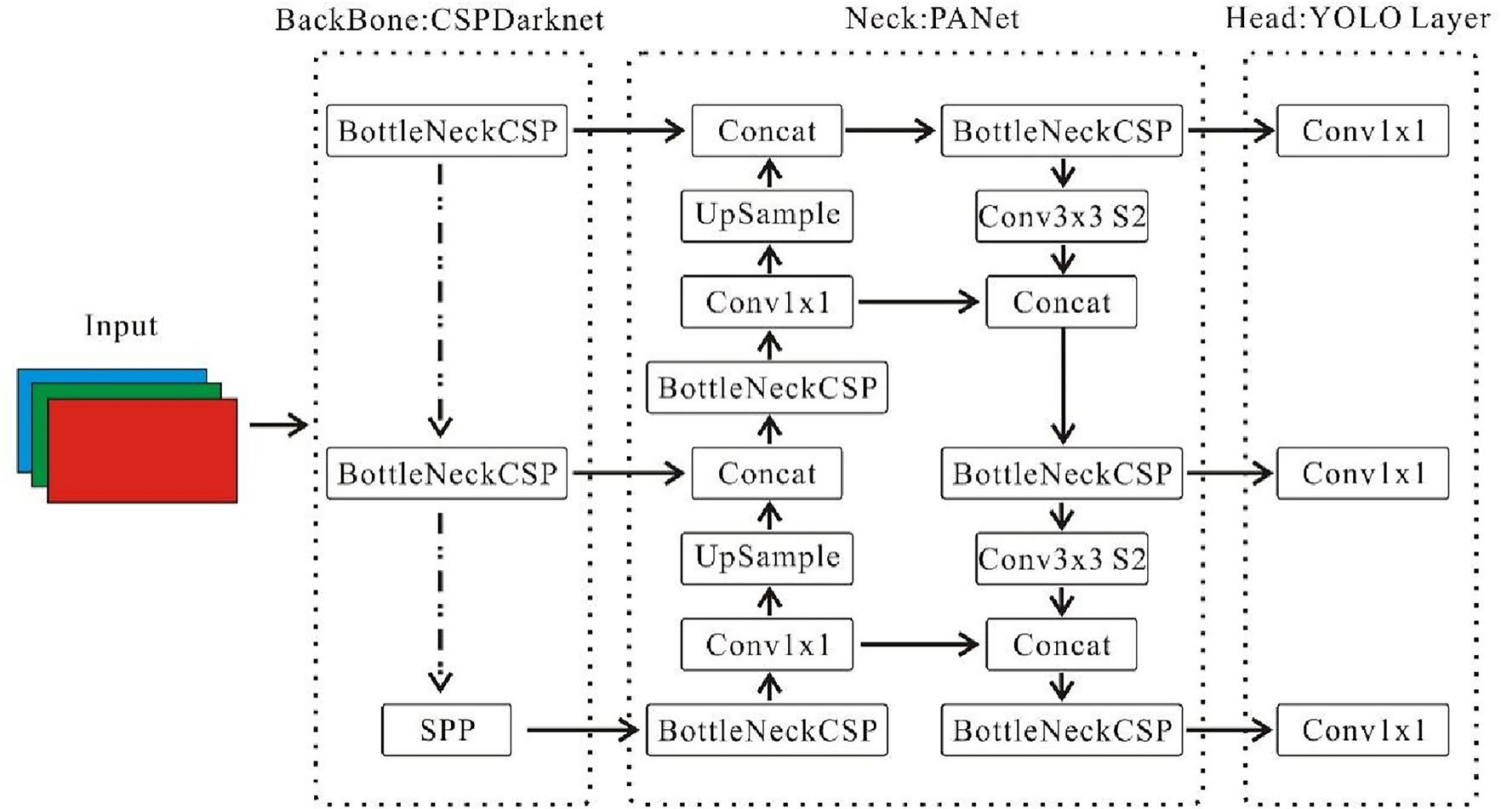
YOLOv5 network architecture.

Building upon the foundation established by its predecessor, YOLOv5 further enhances real-time object detection capabilities through innovative network architecture and strategic integration of advanced techniques. YOLOv5 consists of four main modules in its network architecture, input, backbone, neck, and head, as illustrated in Fig. 4. The input side incorporates strategies such as Mosaic data augmentation, adaptive anchor frame calculation, and adaptive image scaling to enhance performance. Mosaic data augmentation enriches the dataset by randomly scaling, cropping, and arranging multiple images together, significantly bolstering the system's robustness. Adaptive anchor frame calculation and image scaling optimize learning and inference effectiveness. The backbone module employs Focus, Cross Stage Partial Network (CSP), and Spatial Pyramid Pooling (SPP), where the SPP module expands the perceptual field, enhancing target awareness. YOLOv5 introduces Feature Pyramid Network (FPN) + Pyramid Attention Network (PAN) and CSP2 structures in the neck stage, strengthening feature fusion and information transmission for overall performance improvement. In the head stage, Generalized Intersection over Union (GIoU) bounding box loss and weighted Non-Maximum Suppression (NMS) are utilized, optimizing target prediction accuracy and robustness.

### The working principle of the CBAM

**Figure 5:**
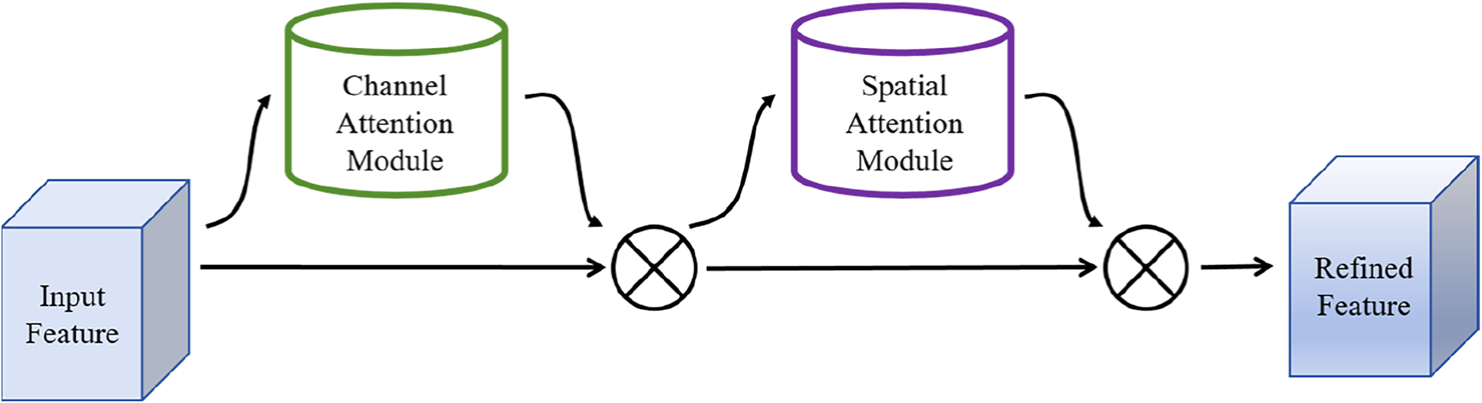
CBAM network structure.

Convolutional Block Attention Mechanism (CBAM) is an attention mechanism designed to enhance the performance of CNN by focusing on important features within the input data^26^. The CBAM module is shown in Fig. 5, which can be primarily divided into two components: the Spatial Attention Module (SAM) and the Channel Attention Module (CAM). In this paper, we enhance the information of interest by introducing the attention mechanism CBAM module after the first convolutional layer of the backbone network. This module assigns weights to feature maps in a learning manner in space and channels, prompting computational resources to be more inclined to the target region of focus, while suppressing useless information.

1. The working principle of spatial attention mechanism.

**Figure 6:**
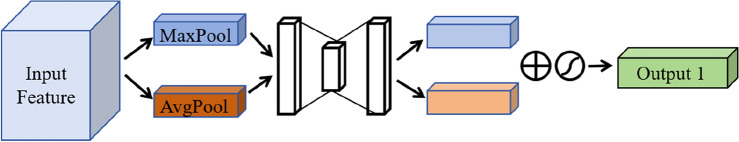
SAM network structure.

The SAM focuses on the importance of each spatial position within the input feature map, as shown in Fig. 6. It begins by performing max-pooling (MaxPool) and average-pooling (AvgPool) on these features in the spatial dimension. The two sets of features obtained after pooling are then separately input into Multilayer Perceptron (MLP) network, where the MLP's role is to facilitate sufficient cross-interaction between feature vectors across different dimensions, capturing more non-linear and composite feature information. Subsequently, the two sets of new features obtained through the MLP network are element-wise added, and finally, through an activation function, a set of desired new features is obtained. This process, by introducing the channel attention mechanism, enables the network to more effectively focus on and integrate features, thereby improving model performance.

2. The working principle of channel attention mechanism.

**Figure 7:**
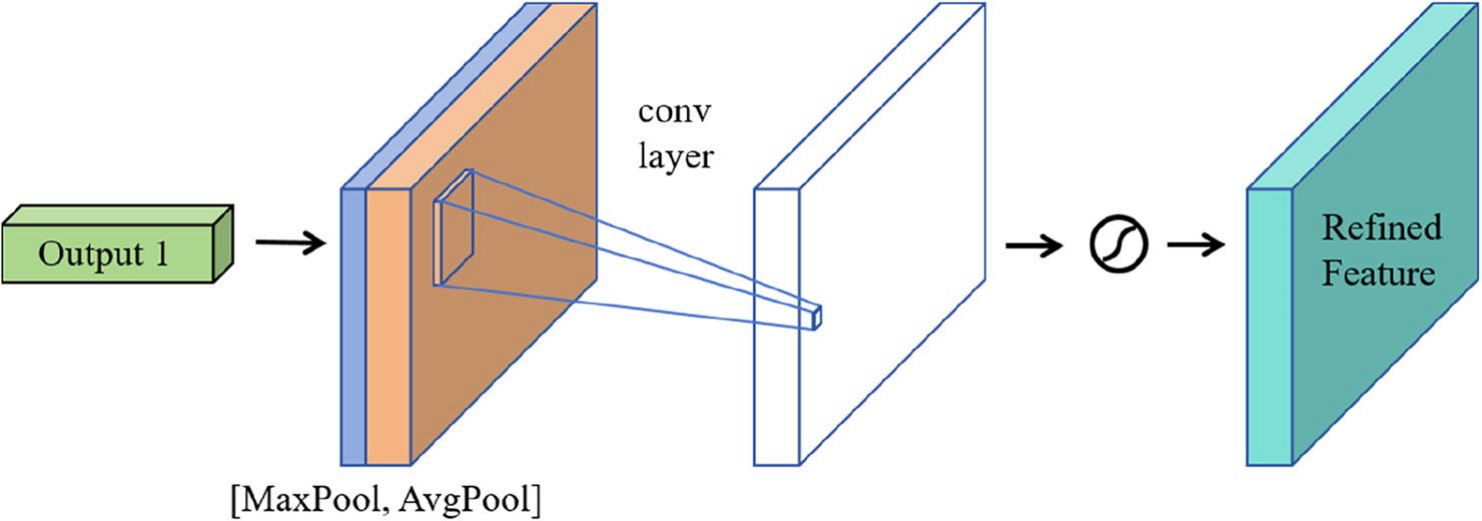
CAM network structure.

The CAM focuses on the importance of each channel for the final output as shown in Fig. 7. The process involves taking a set of features as input and then operating on each channel individually. This includes applying global max pooling and global average pooling to each channel. Subsequently, the results of these two pooling operations are concatenated to form new features. These new features undergo further processing, typically involving convolutional operations, to generate a 2D spatial attention feature map. Finally, the obtained features are processed through an activation function to achieve the desired representation. This process enables the model to better understand the importance of each channel, thereby enhancing the overall performance of the network.

### Improved YOLOv5 algorithm

**Figure 8:**
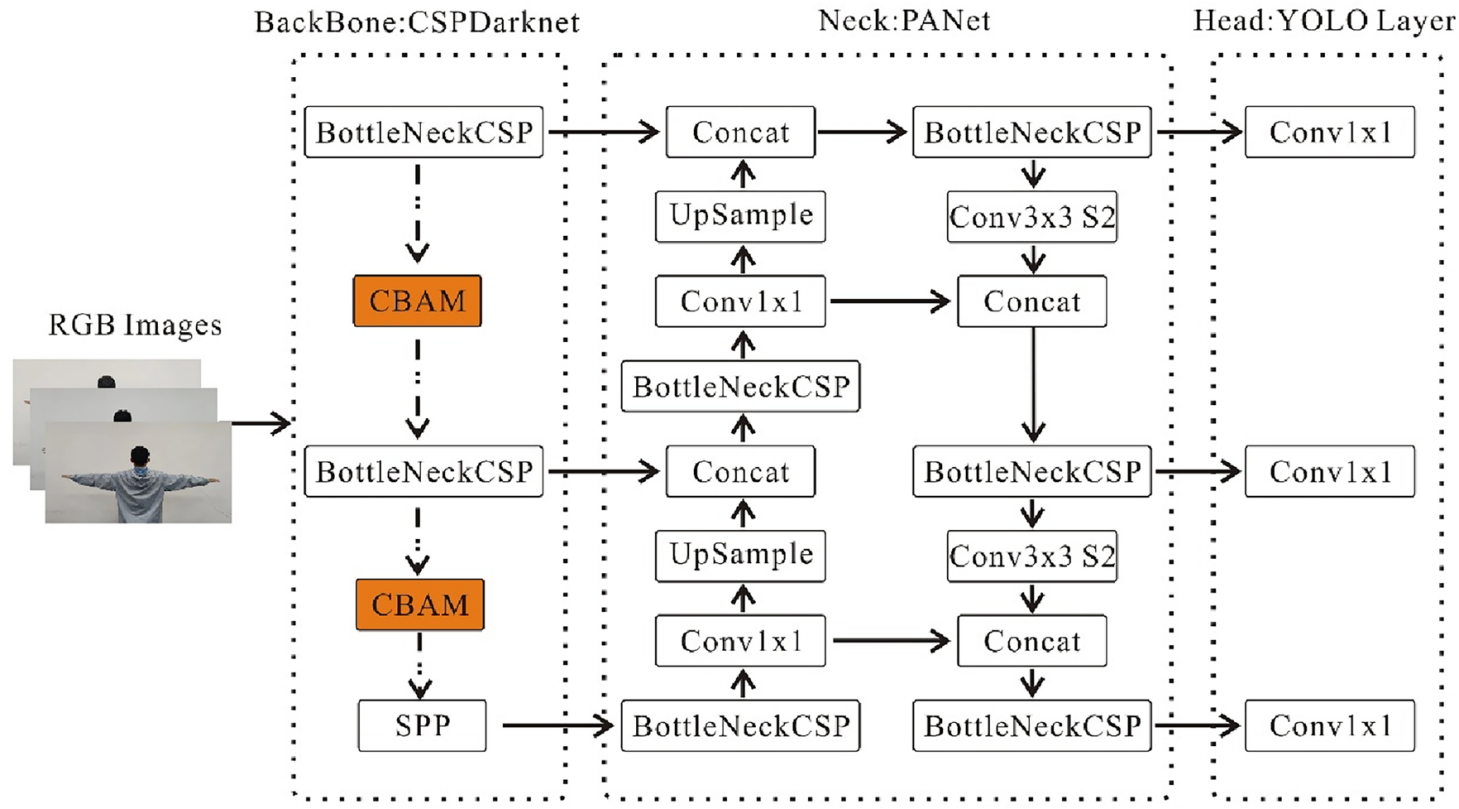
Improve YOLOv5 network architecture.

To address the issue of weak extraction of pose defect feature information, the introduction of the CBAM module in the backbone part of YOLOv5 effectively enhances the model's capability to extract features from different types of defective channels and spatial information. This enhancement significantly improves the detection capability of the model. The improved detection framework of YOLOv5 algorithm is shown in Fig. 8.

### Posture estimation for Asian people

In evaluating the quality of a human pose estimation library, assessment metrics play a vital role^27^. The evaluation metric used in this study is the percentage of detected joints (*PDJ*), which measures the performance of Asian human pose estimation libraries. *PDJ* employs the Euclidean distance between ground truth and predicted keypoints to assess the detection accuracy of Asian human pose estimation libraries. The higher the value of *PDJ*, the higher the accuracy rate. The calculation of Euclidean distance $d(x,y)$ between the ground truth $({x_1},{y_1})$ and predicted keypoints $({x_2},{y_2})$ is shown in Eq. (1).

1 $$d\left( {x,y} \right) = \sqrt {{{\left( {{x_1} - {x_2}} \right)}^2} + {{\left( {{y_1} - {y_2}} \right)}^2}}$$

The threshold of *PDJ* was 0.05 for the value of the torso diameter. The torso diameter (*TD*) was computed for the Euclidean distance from the left shoulder to the right hip, represented as the coordinates $({x_{ls}},{y_{ls}})$ and $({x_{rh}},{y_{rh}})$, as shown in Eq. (2).

2 $$TD = \sqrt {{{\left( {{x_{ls}} - {x_{rh}}} \right)}^2} + {{\left( {{y_{ls}} - {y_{rh}}} \right)}^2}}$$

When the distance between the predicted keypoints and ground truth keypoints was smaller than the threshold, the predicted keypoints were considered to be correctly detected. In this scenario, the $bool$ will be regarded as true or fulfilled; otherwise, it will be deemed as false. Hence, the *PDJ* can be deduced as shown in Eq. (3), where n represents the total number of predicted joints.

3 $$PDJ = \frac{{\sum\nolimits_{i = 1}^n {boo} l({d_i} \times TD)}}{n}$$

### Ethics approval and consent to participate

This study involves the use of a range of motion assessment system for spinal disorders and frozen shoulder that combines MediaPipe and YOLOv5. We hereby state that we have obtained informed consent from all subjects and/or their legal guardians to release information and images in online open access publications that may lead to their identification. The experiments were conducted in accordance with relevant guidelines and regulations, and approval was obtained from the Ethics Committee of Shaoxing People's Hospital. We confirm that informed consent was obtained from all participants.

## Experimental design

### Deployment of C/S architecture in terms of software and hardware

**Figure 9:**
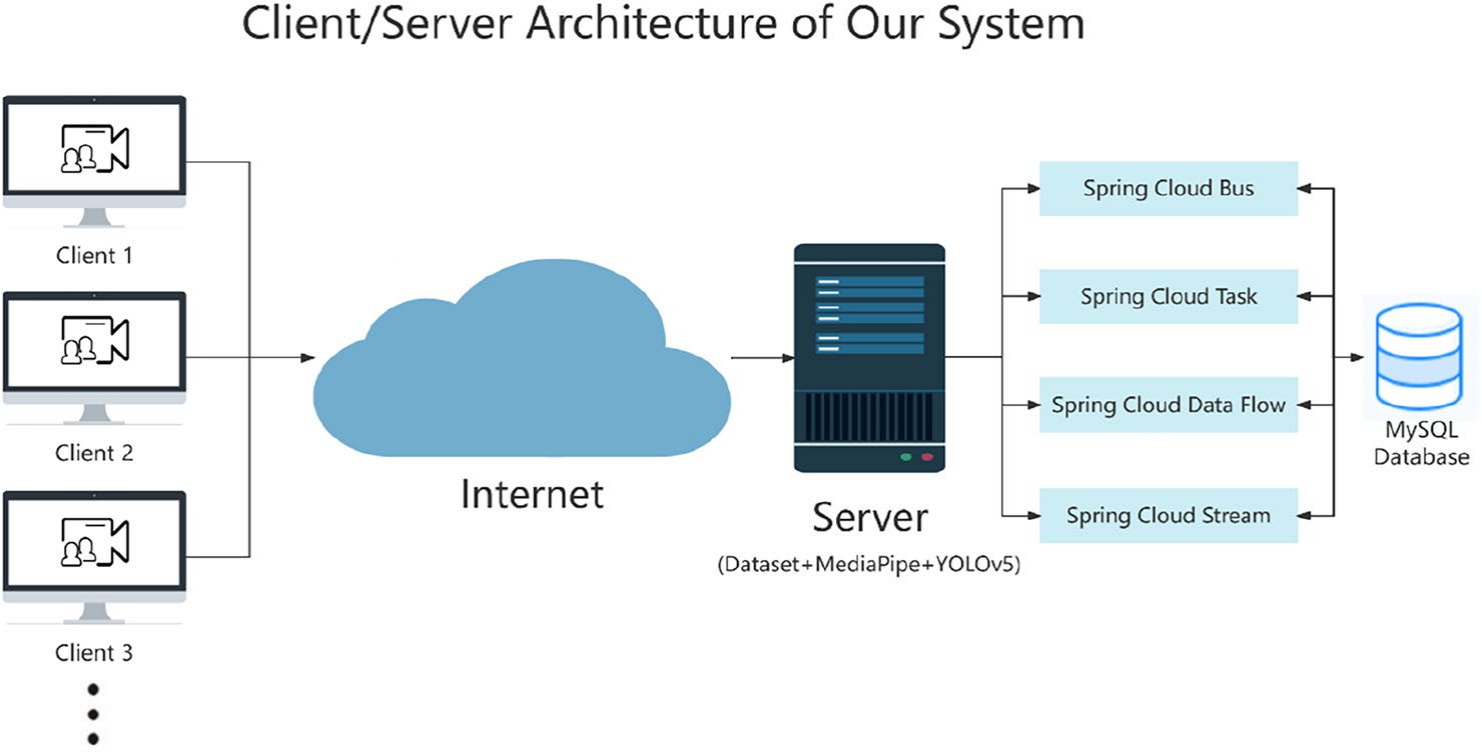
The working principle of our C/S system.

To fulfill large-scale computing needs, it is crucial to adopt a client/server (C/S) architecture^28^. A spine disease and frozen shoulder assessed system based on C/S architecture is an innovative medical tool, as shown in Fig. 9. On the client side, users can easily upload image or video data and achieve real-time data transmission with the server side through the WebSocket communication protocol. Additionally, on the server side, YOLOv5 and MediaPipe models are equipped to process the data, and multiple components of Spring Cloud are integrated to enhance the flexibility and scalability of the system. Spring Cloud provides a comprehensive solution for microservices architecture, enhancing data processing and communication efficiency. It integrates message passing, task execution, data stream management, and configuration synchronization to ensure the system's orderliness, consistency, and rapid response capability. With the support of Spring Cloud, the system's scalability and flexibility are further enhanced, enabling support for complex interactions and dynamic configuration management between services. With the support of Spring Cloud components, we efficiently manage information using MySQL 8.0.26 to ensure data persistence and secure storage.

In C/S architecture, deploying both the client and the server involves several stages including hardware setup, operating system installation, application setup, and database management system configuration. These stages require meticulous planning and management to ensure the high performance and reliability of the C/S system, as shown in Table 1.

**Table 1:** Software and hardware deployment.

Deployment	Client	Server
Hardware	The client devices can be desktop computers, or smartphones, but must have a camera	Dell PowerEdge R740 is equipped with an Xeon 5218R processor, 128GB of memory, and RTX 3090 Ti
Operating system	Windows(7/8/10/11), Android(10/11/12)	Linux(Centos 7.5)
Application installation	Install and configure applications to ensure compatibility with the server	PyCharm, Unity and Visual Studio serve as the primary development tool

### Data collection

Spinal diseases and frozen shoulder are conditions that involve a variety of information. For spinal disease evaluations, this typically involves measuring the curvature metrics of the patient's spine, performing a bone density analysis, and detecting the presence of any abnormalities or injuries. In contrast, range of motion assessment for frozen shoulder may involve measuring the mobility of the shoulder joint, assessing muscle tone, and determining the position of the scapula, among other metrics. During data collection, this study will focus on spinal curvature, shoulder range of motion, and range of motion metrics associated with frozen shoulder.

**Figure 10:**
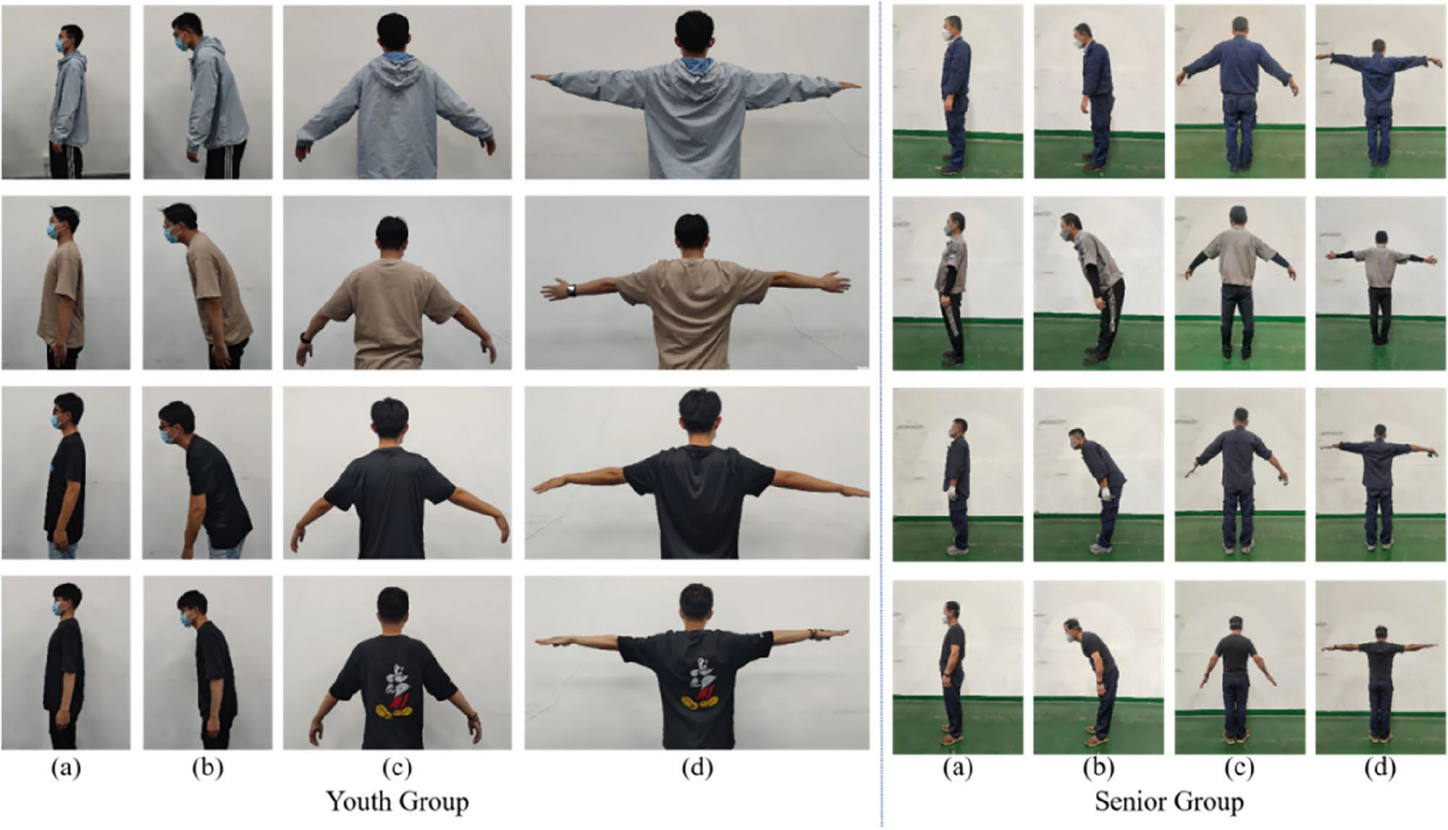
Human posture datasets: (**a**) Upright posture datasets; (**b**) Hunchbacked posture datasets; (**c**) Bent posture datasets; (**d**) Extended posture datasets.

In this study, the collection of data sets was assisted and guided by Shaoxing Hospital’s physicians with extensive clinical experience to ensure the professionalism and accuracy of the data. These physicians collected relevant information through clinical observation and functional testing. During the data collection process, the physicians were able to provide professional guidance to ensure that the process of data collection complied with standard operating procedures to ensure the reliability and accuracy of the data. Each action posture was defined by a set of angles formed between joints. These individual angles were used to determine the correct positioning of action postures^29^. The reference data for the following action postures were included in the database. Sample human posture images are presented in Fig. 10, encompassing images of different action postures such as upright, hunchbacked, bent, and extended postures.

### Parameter configuration

In our model, a series of key parameters are configured to optimize the learning and convergence performance of the model^30^. The initial learning rate was set to 0.01 to ensure that in the early stages of training, the model uses relatively small step sizes to update the weights, thus stabilizing the training process. As training progresses, the learning rate is gradually reduced to a final value of 0.2 to further tune the model parameters and improve stability. In order to accelerate the convergence of the model, the Stochastic Gradient Descent (SGD) momentum was set to 0.937. Additionally, a weight decay factor of 0.0005 was introduced to impose an additional penalty during the weight updating process to reduce the risk of over fitting. The combined effect of these parameter configurations ensured that the model was able to enter training smoothly and achieve good learning results.

### Image annotation method for motion detection

Motion detection is a supervised learning task^31^. Model training requires information about the classification and location of actions in the images. In terms of action target classification, the practical application of action target detection in indoor scenes is considered. Based on the images captured by the camera, action classes are differentiated in the images^32^. The target motions are divided into four categories, such as ‘Hunchback’, ‘Stand’, ‘Stretch’ and ‘Bend’ in Table 2.

**Table 2:** YOLO annotation category information.

Number	Category	Image count
0	Hunchback	134
1	Stand	120
2	Stretch	112
3	Bend	113

Data annotation is a tedious and repetitive task that must be performed manually. A portion of the image samples was annotated with bounding boxes by using the object detection and annotation tool Labelimg^33^. Then, in the YOLO annotation format txt file, each line represents the category and location information of an object^34^. The first column indicates the object’s category, followed by four columns that represent the object’s position information, namely X, Y, W, and H. Each image has a corresponding txt file, and a single file can contain multiple objects of different categories. In this format, X and Y represent the center coordinates of the object, while W and H are normalized using the original image width^35^.

Validating annotated data is a crucial step to ensure accuracy and reliability. We employ a cross-validation approach, relying on multiple experienced medical professionals to independently annotate the same data. Their annotations are then compared and verified. By comparing the annotations from different annotators, potential errors or inconsistencies can be promptly identified and corrected, thereby enhancing the accuracy and consistency of the annotations. This validation process not only helps reduce human annotation errors but also improves the overall data quality, providing a reliable foundation for subsequent medical research and applications.

## Experiments results

### Comparison of object detection models train

R-CNN, Fast R-CNN, Faster R-CNN, and YOLOv5 stand as classical algorithms in the field of object detection, each appearing at different points in time and driving the progress of this field. R-CNN introduced the concept of region proposal extraction, enabling the use of CNN for object detection. Fast R-CNN further enhanced the speed and accuracy of R-CNN, introducing the ROI pooling layer, although it still required a two-step training process. Faster R-CNN brought forth the RPN, achieving end-to-end object detection and marking a significant milestone in the field. YOLOv5, on the other hand, is an efficient object detection algorithm with outstanding real-time performance, suitable for various applications. These models yield varied results in the detection of spinal diseases and frozen shoulder, as illustrated in Table 3.

**Table 3:** Comparison of object detection models.

Models	mAP%	Detection speed (ms)	Real-time detection
Fast R-CNN	86.4	320ms per image	No
Faster R-CNN	88.4	198ms per image	Yes
YOLOv5	90.3	35ms per image	Yes

### Performance comparison between YOLOv5 and MediaPipe + YOLOv5

**Figure 11:**
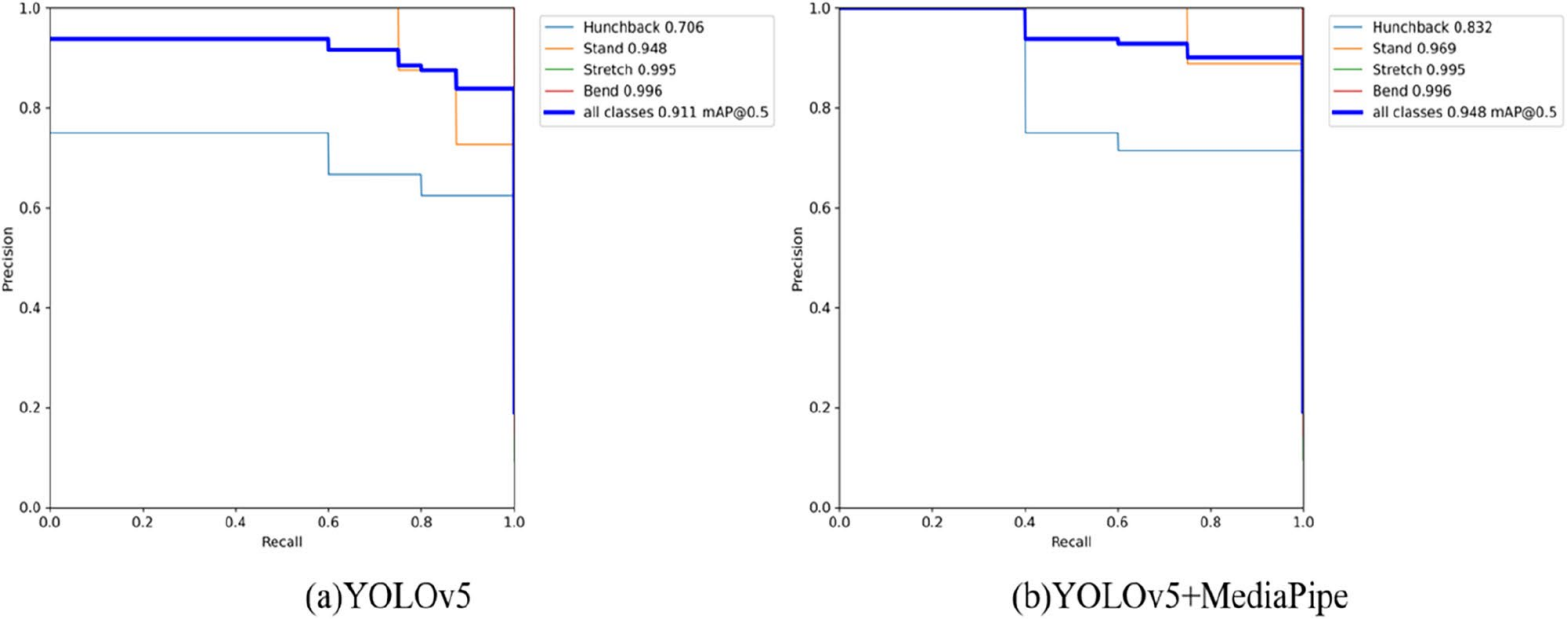
Comparison of the PR curves between YOLOv5 and MediaPipe + YOLOv5.

In order to perform a comprehensive investigation and comparison of the performance between YOLOv5 and MediaPipe + YOLOv5 in the realm of detecting spinal diseases and frozen shoulder, we conducted a series of experiments. The results before and after algorithm enhancements are depicted in Fig. 11. The Precision-Recall (PR) curve is a crucial tool for evaluating model performance. Precision indicates the proportion of correctly predicted positive instances among all predicted positives, while recall measures the proportion of actual positive instances successfully predicted by the model. In the PR curve, an upward trend towards the right signifies that the model can capture more positives while maintaining high precision. When the PR curve approaches the upper-left corner, it indicates that the model maintains high precision even under high recall conditions. Therefore, the combination of Mediapipe and YOLOv5 yields superior model performance.

Additionally, we integrated the YOLOv5 model into a cross-platform application via the MediaPipe framework to expand its deployment capabilities. Comprehensive experiments involved the training and validation, focusing on metrics such as recall, precision, mAP and model size. Based on the comparative analysis of experimental results, as shown in Table 4. MediaPipe + YOLOv5 performs better than using YOLOv5 alone in performance metrics such as ‘Precision’, ‘Recall’, and ‘mAP’.

**Table 4:** Experimental results of different modules on our datasets.

Method	Precision/%	Recall%	mAP/%	Model size/MB
YOLOv5	81.2	85.5	90.3	167
MediaPipe + YOLOv5	95.3	90.2	94.6	262.3

### Experimental results of YOLOv5 and MediaPipe

The improved YOLOv5 object detection combined with MediaPipe for extracting human keypoints and skeleton information was used for human pose recognition, with validation results detailed in Table 5. The detection performance was evaluated for four different poses: hunching, standing, stretching and bending. The results showed that the precision and recall were as high as 92.3–98.7% and the mAP ranged from 90.4 to 98.6%, respectively. These data demonstrate the excellent performance of the method for range of motion assessment.

**Table 5:** Detection results of MediaPipe and YOLOv5 on our datasets.

Class	Precision/%	Recall/%	mAP/%
Hunchback	92.3	88.3	90.4
Stand	92.9	95.6	95.5
Stretch	96.6	97.2	98.6
Bend	98.7	99.6	98.2

**Figure 12:**
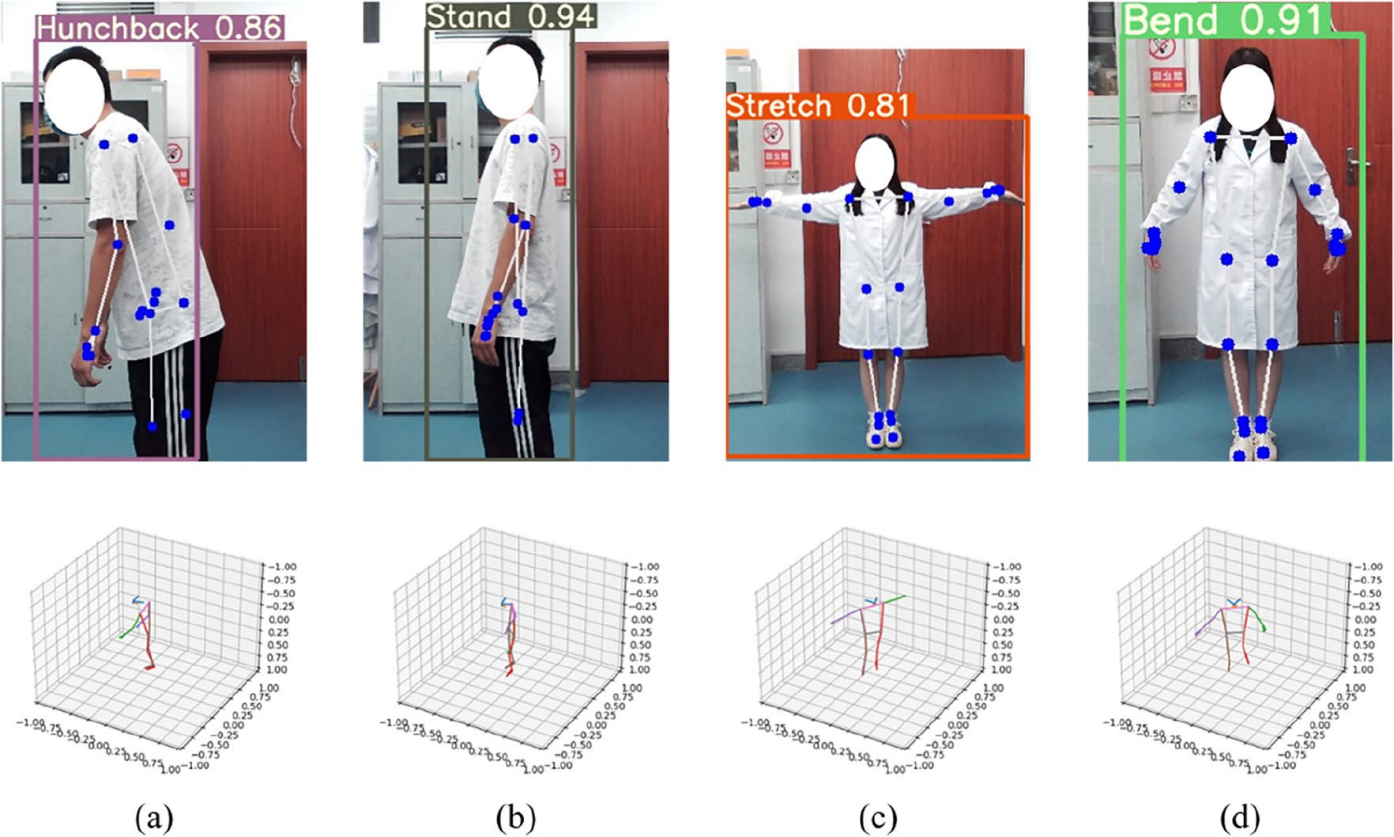
Motion poses and feedback results: (**a**) Judge the hunchback posture; (**b**) Judge the stand posture; (**c**) Judge the stretch posture; (**d**) Judge the bend posture.

In the study, patients were invited to perform a series of specific actions or poses, and their motion data were recorded and analyzed using the system, as shown in Fig. 12. By combining target detection and bone tracking technology, the system utilizes YOLOv5 for target detection, which can quickly and accurately identify target objects and mark their poses in the bounding box, which provides the basis for subsequent analysis. Then using MediaPipe for bone tracking, the keypoints and skeletal structures of the human body can be more finely acquired to further analyze the accuracy and smoothness of the motion poses.

### Spinal disease assessment experiments

**Figure 13:**
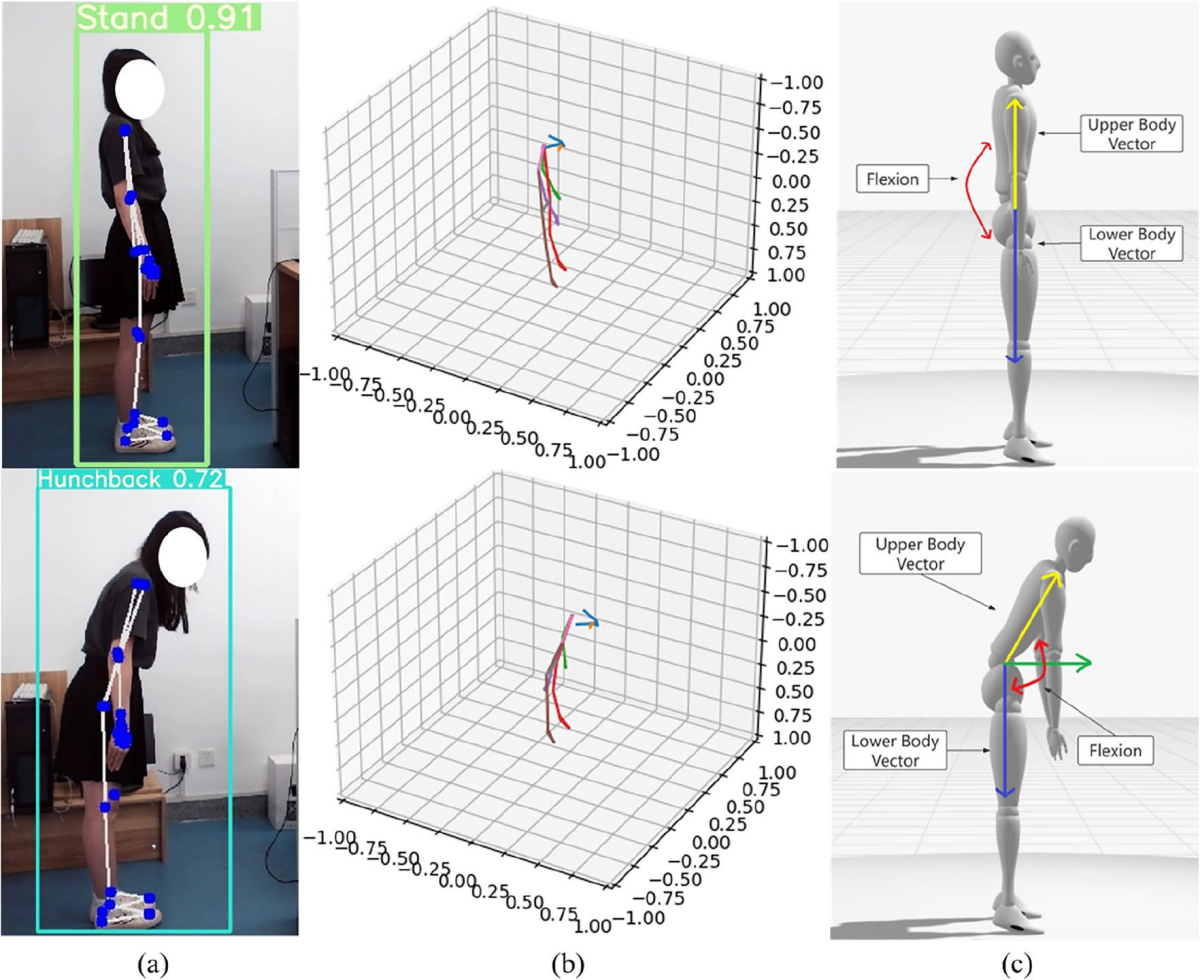
Spine diseases assessment: (**a**) Spinal disease result images generated by combining YOLOv5 and MediaPipe; (**b**) Human skeletal structure generated based on keypoint information extracted by MediaPipe; (**c**) Calculation of spinal range of motion angles by projecting angles on defined anatomical planes.

Combining YOLOv5 and MediaPipe technologies enables real-time tracking and analysis of spinal curvature, promptly identifying potential abnormalities or trends changes, and providing vital insights for the prevention and treatment of spinal diseases. The effectiveness of the testing conducted on subjects is illustrated in Fig. 13. In the experiment, YOLOv5 is utilized for spinal diseases detection, such as upright posture and hunchback. Subsequently, MediaPipe is employed to extract keypoints and skeletal information, such as spinal bending and twisting movements. By integrating these two technologies, we can obtain more comprehensive and accurate information about spinal disorders, addressing patients' range of motion and potential pathological conditions.

### Frozen shoulder assessment experiments

**Figure 14:**
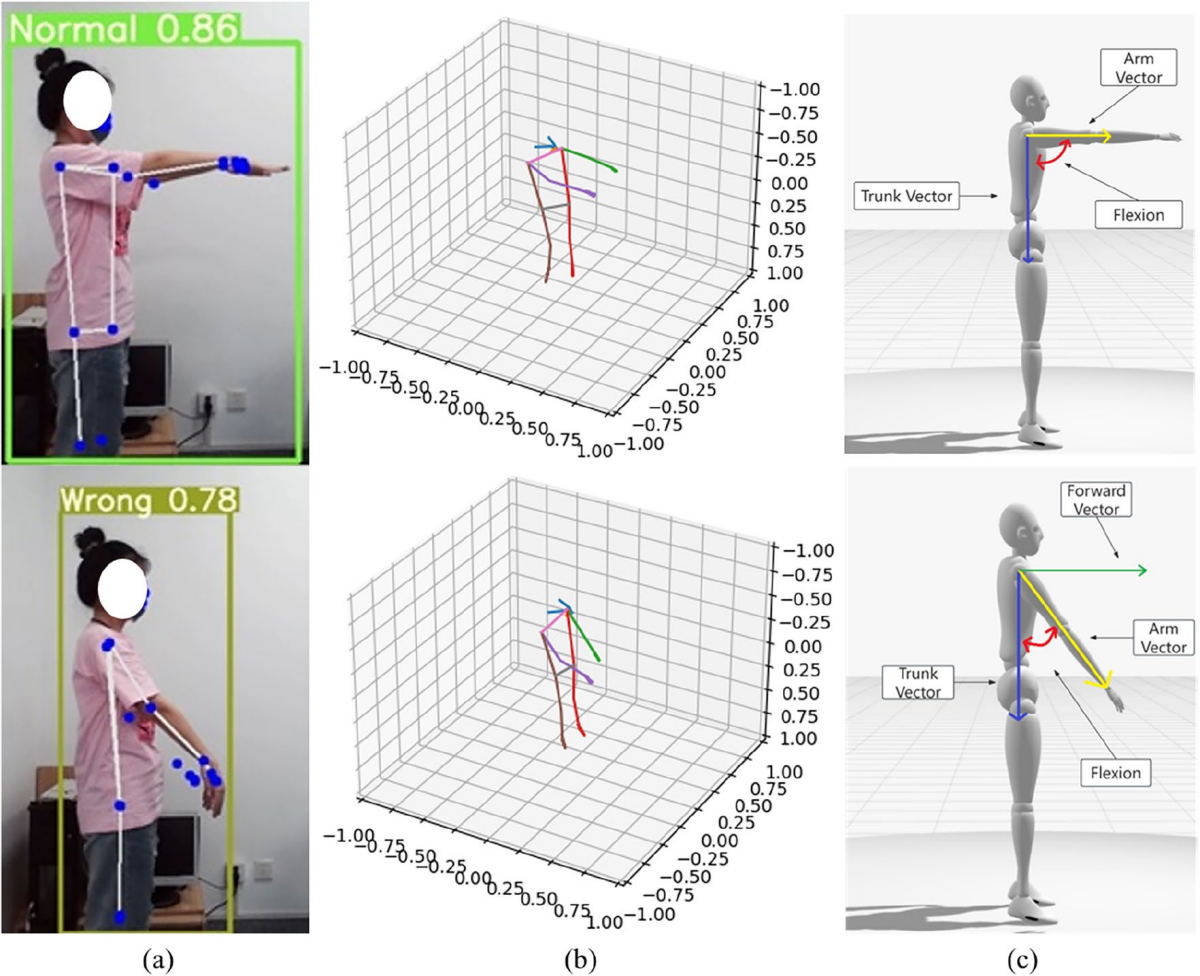
Frozen shoulder assessment: (**a**) Frozen shoulder result images generated by combining YOLOv5 and MediaPipe; (**b**) Human skeletal structure generated based on keypoints information extracted by MediaPipe; (**c**) Calculation of frozen shoulder range of motion angles by projecting angles on defined anatomical planes.

During the assessment of frozen shoulder range of motion, we can refer to the experimental results, as detailed in Fig. 14. In the experiment, we first utilized YOLOv5 for the detection of frozen shoulder, which includes assessing the forward bending situation of patients, including normal and restricted forward bending, as well as the range of motion. Subsequently, we employed MediaPipe technology to extract keypoints and skeletal information. By integrating these two technologies, we can obtain more comprehensive and accurate information about frozen shoulder, thus better addressing patients' range of motion issues and potential pathological conditions.

## Discussion

### The application of visual analog scale in frozen shoulder assessment

In the assessment of frozen shoulder, it is crucial to consider both frozen shoulder and visual analog scale (VAS) scores comprehensively. Besides evaluating the level of pain, it is also necessary to consider the functional impairment of the shoulder joint and the limitation of motion range. Physicians assess the systemic range of motion of the shoulder joint to identify symptoms and understand their impact on the patient's mobility. The basis and criteria for assessment in VAS is usually a specific attribute or state of the subject being assessed^36^. This may involve subjective feelings in terms of pain levels, satisfaction, anxiety levels, etc. Criteria are usually provided by the researcher and can be definitions and descriptions of the assessed attribute or state to help the person being assessed understand the assessment.

In this study, we first utilized the frozen shoulder range of motion assessment system based on MediaPipe and YOLOv5 to interpret the forward bending situation and range of motion of the evaluated subjects. Subsequently, based on the subjects' conditions, we quantified the level of pain into four grades ranging from mild to severe and conducted VAS assessment scoring on the evaluated subjects. The VAS score serves as a crucial tool for quantifying the degree of pain, aiding physicians in understanding the patient's pain condition, and guiding the formulation of treatment plans while monitoring their effectiveness. As shown in Table 6, the VAS score statistics of participants are provided, where a VAS score of 0 indicates no pain, 1–3 points indicate mild pain, 4–7 points indicate moderate pain, and 8–10 points indicate severe pain. Therefore, considering both the frozen shoulder motion range assessment and VAS scores can offer a more comprehensive and accurate patient evaluation for personalized treatment plans.

**Table 6:** VAS score statistics of participants.

Participants number	Frozen shoulder assessment system	Pain level	VAS score
1	Restricted motion	Moderate pain	6
2	Normal motion	Mild pain	3
3	Normal motion	Mild pain	2
4	Normal motion	Mild pain	2
5	Restricted motion	Moderate pain	6

### The "Picking Bayberries" game development

**Figure 15:**
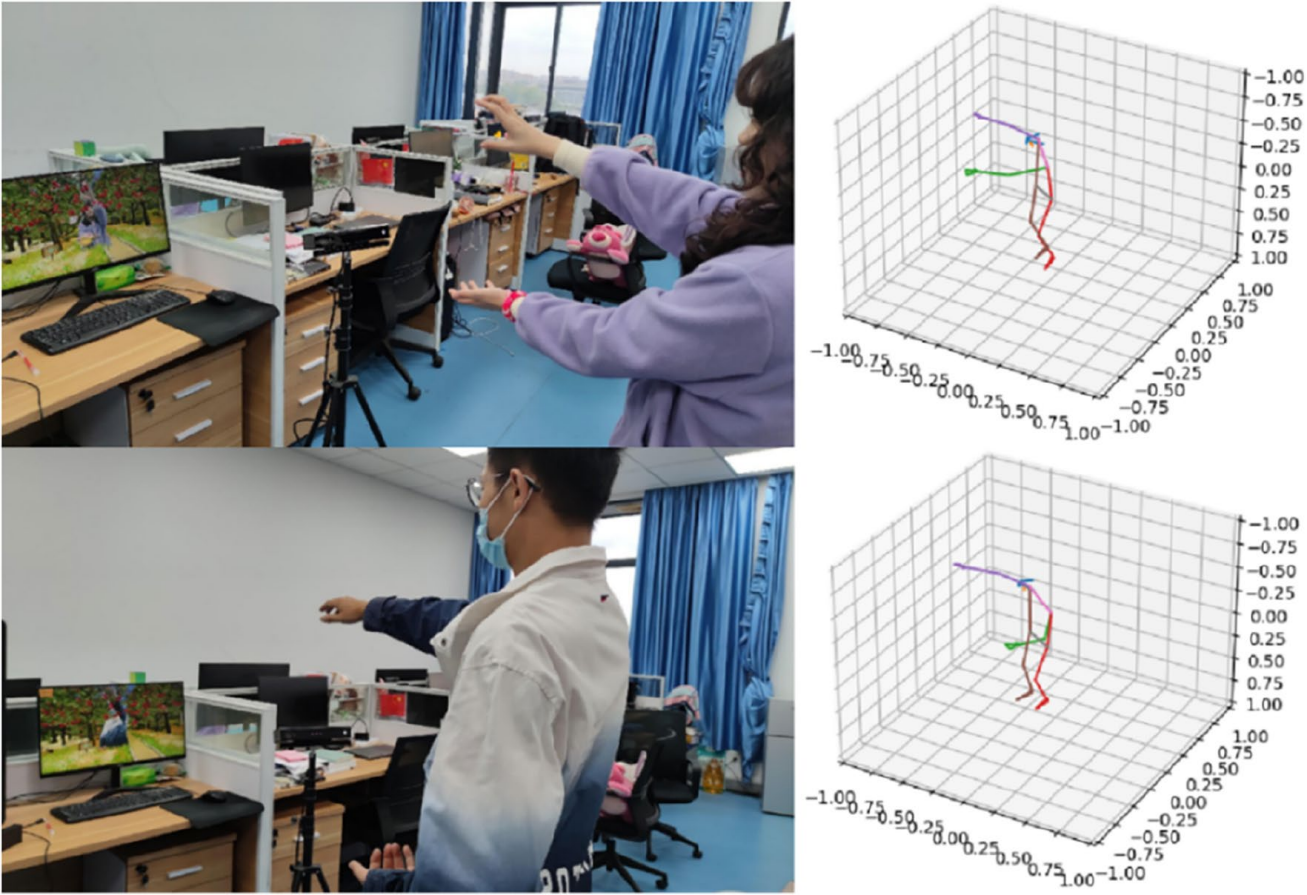
Rehabilitation train through picking bayberries game.

This study has designed an application called the “Picking Bayberries” game to aid patients with spinal disorders and frozen shoulder in their exercise and rehabilitation routines. This game integrates the range of motion assessment system of MediaPipe and YOLOv5, thus enabling the real-time analysis of users’ movements and providing personalized exercise plans for them. Users need to follow real-time feedback and guidance from the system to perform moderate bending of the waist and flexing of the arms to successfully pick the virtual mulberries, as shown in Fig. 15. These actions can help exercise and strengthen the muscles of the back and shoulders, thus enhancing the rehabilitation and improvement of individuals with spinal diseases and frozen shoulder. The mechanism of this application involves utilizing MediaPipe and YOLOv5 technologies to analyze user movements in real-time. The system provides feedback based on the quality of user movements to assist them in performing actions correctly. Through this approach, it helps alleviate physical pain, improve poor posture, and enhance functional mobility in daily life.

## Conclusion

This study has pioneered the development of a real-time motion assessment system, a pioneering integration of MediaPipe's pose estimation and YOLOv5's object detection algorithms. The system exhibits remarkable precision and reliability in the complex domain of posture analysis and range of motion assessment. It meticulously records and analyzes patients' postures and movements, equipping healthcare professionals with a powerful tool for a comprehensive evaluation of spinal diseases and frozen shoulder. As our system continues to evolve, we anticipate a substantial enhancement in the well-being and quality of life for individuals grappling with spinal and shoulder health challenges.

## Data Availability

The datasets used and analyzed during the current study are available from the corresponding author upon request.

## References

[1] Safiri S (2020). Global, regional, and national burden of neck pain in the general population, 1990–2017: Systematic analysis of the Global Burden of Disease Study 2017. BMJ.

[2] Da Costa RC, Moore SA (2010). Differential diagnosis of spinal diseases. Vet. Clin. Small Anim. Pract..

[3] Millar NL (2022). Frozen shoulder. Nat. Rev. Dis. Primer.

[4] Xu, J. *et al.* Deep kinematics analysis for monocular 3d human pose estimation. In *Proceedings of the IEEE/CVF Conference on Computer Vision and Pattern recognition* 899–908 (2020).

[5] Li, J. *et al.* Hybrik: A hybrid analytical-neural inverse kinematics solution for 3d human pose and shape estimation. In *Proceedings of the IEEE/CVF Conference on Computer Vision and Pattern Recognition* 3383–3393 (2021).

[6] Zhang, Y. Applications of google MediaPipe pose estimation using a single camera. (2022).

[7] Kim J-W, Choi J-Y, Ha E-J, Choi J-H (2023). Human pose estimation using mediapipe pose and optimization method based on a humanoid model. Appl. Sci..

[8] Anilkumar, A., Athulya, K. T., Sajan, S. & Sreeja, K. A. Pose estimated yoga monitoring system. In *Proceedings of the International Conference on IoT Based Control Networks & Intelligent Systems-ICICNIS* (2021).

[9] Girshick, R., Donahue, J., Darrell, T. & Malik, J. Rich feature hierarchies for accurate object detection and semantic segmentation. In *Proceedings of the IEEE Conference on Computer Vision and Pattern Recognition* 580–587 (2014).

[10] Girshick, R. Fast r-cnn. In *Proceedings of the IEEE International Conference on Computer Vision* 1440–1448 (2015).

[11] Ren, S., He, K., Girshick, R. & Sun, J. Faster r-cnn: Towards real-time object detection with region proposal networks. *Adv. Neural Inf. Process. Syst.* **28**, (2015). 10.1109/TPAMI.2016.2577031 27295650

[12] Zhan W (2022). An improved Yolov5 real-time detection method for small objects captured by UAV. Soft Comput..

[13] Redmon, J., Divvala, S., Girshick, R. & Farhadi, A. You only look once: Unified, real-time object detection. In *Proceedings of the IEEE Conference on Computer Vision and Pattern Recognition* 779–788 (2016).

[14] Redmon, J. & Farhadi, A. YOLO9000: Better, faster, stronger. In *Proceedings of the IEEE Conference on Computer Vision and Pattern Recognition* 7263–7271 (2017).

[15] Redmon, J. & Farhadi, A. Yolov3: An incremental improvement. ArXiv Prepr. ArXiv180402767 (2018).

[16] Bochkovskiy, A., Wang, C.-Y. & Liao, H.-Y. M. Yolov4: Optimal speed and accuracy of object detection. ArXiv Prepr. ArXiv200410934 (2020).

[17] Song Q (2021). Object detection method for grasping robot based on improved YOLOv5. Micromachines.

[18] Chen R, Tian X (2023). Gesture detection and recognition based on object detection in complex background. Appl. Sci..

[19] Nguyen H-C (2022). Combined YOLOv5 and HRNet for high accuracy 2D keypoint and human pose estimation. J. Artif. Intell. Soft Comput. Res..

[20] Mou F, Ren H, Wang B, Wu D (2022). Pose estimation and robotic insertion tasks based on YOLO and layout features. Eng. Appl. Artif. Intell..

[21] Du Q, Bai H, Zhu Z (2023). Intelligent evaluation method of human cervical vertebra rehabilitation based on computer vision. Sensors.

[22] Garg S, Saxena A, Gupta R (2022). Yoga pose classification: A CNN and MediaPipe inspired deep learning approach for real-world application. J. Ambient Intell. Humaniz. Comput..

[23] Latreche A, Kelaiaia R, Chemori A, Kerboua A (2023). Reliability and validity analysis of MediaPipe-based measurement system for some human rehabilitation motions. Measurement.

[24] Pestana D (2021). A full featured configurable accelerator for object detection with YOLO. IEEE Access.

[25] Chen, S. & Chen, B. Research on object detection algorithm based on improved Yolov5. In *Artificial Intelligence in China: Proceedings of the 3rd International Conference on Artificial Intelligence in China* 290–297 (Springer, 2022).

[26] Woo, S., Park, J., Lee, J.-Y. & Kweon, I. S. Cbam: Convolutional block attention module. In *Proceedings of the European Conference on Computer Vision (ECCV)* 3–19 (2018).

[27] Chung J-L, Ong L-Y, Leow M-C (2022). Comparative analysis of skeleton-based human pose estimation. Future Internet.

[28] Xue, M. & Zhu, C. The socket programming and software design for communication based on client/server. In *2009 Pacific-Asia Conference on Circuits, Communications and Systems* 775–777 (IEEE, 2009).

[29] Lv Z, Penades V, Blasco S, Chirivella J, Gagliardo P (2016). Evaluation of Kinect2 based balance measurement. Neurocomputing.

[30] Zhang Y (2022). Real-time vehicle detection based on improved yolo v5. Sustainability.

[31] Azam, M., Blayo, M., Venne, J.-S. & Allegue-Martinez, M. Occupancy estimation using wifi motion detection via supervised machine learning algorithms. In *2019 IEEE Global Conference on Signal and Information Processing (GlobalSIP) *1–5 (IEEE, 2019).

[32] Ivasic-Kos, M., Kristo, M. & Pobar, M. Person detection in thermal videos using YOLO. In *Intelligent Systems and Applications: Proceedings of the 2019 Intelligent Systems Conference (IntelliSys)*, vol. 2, 254–267 (Springer, 2020).

[33] Wang K (2019). Perspective transformation data augmentation for object detection. IEEE Access.

[34] Shinde S, Kothari A, Gupta V (2018). YOLO based human action recognition and localization. Proc. Comput. Sci..

[35] Mamdouh N, Khattab A (2021). YOLO-based deep learning framework for olive fruit fly detection and counting. IEEE Access.

[36] Shirahama N, Watanabe S, Moriya K, Koshi K, Matsumoto K (2021). A new method of subjective evaluation using visual analog scale for small sample data analysis. J. Inf. Process..

